# Properties of the Visible Light Phototaxis and UV Avoidance Behaviors in the Larval Zebrafish

**DOI:** 10.3389/fnbeh.2016.00160

**Published:** 2016-08-19

**Authors:** Drago A. Guggiana-Nilo, Florian Engert

**Affiliations:** ^1^Graduate Program in Biophysics, Harvard University, CambridgeMA, USA; ^2^Department of Molecular and Cellular Biology, Harvard University, CambridgeMA, USA

**Keywords:** larval zebrafish, ultraviolet light, vision, phototaxis, color

## Abstract

For many organisms, color is an essential source of information from visual scenes. The larval zebrafish has the potential to be a model for the study of this topic, given its tetrachromatic retina and high dependence on vision. In this study we took a step toward understanding how the larval zebrafish might use color sensing. To this end, we used a projector-based paradigm to force a choice of a color stimulus at every turn of the larva. The stimuli used spanned most of the larval spectral range, including activation of its Ultraviolet (UV) cone, which has not been described behaviorally before. We found that zebrafish larvae swim toward visible wavelengths (>400 nm) when choosing between them and darkness, as has been reported with white light. However, when presented with UV light and darkness zebrafish show an intensity dependent avoidance behavior. This UV avoidance does not interact cooperatively with phototaxis toward longer wavelengths, but can compete against it in an intensity dependent manner. Finally, we show that the avoidance behavior depends on the presence of eyes with functional UV cones. These findings open future avenues for studying the neural circuits that underlie color sensing in the larval zebrafish.

## Introduction

The ability to detect and use colors is ubiquitous in nature, and is an important element of feature detection in visual scenes (Kelber et al., 2003). Color vision begins with the wavelength selective absorption of light by the eye’s photoreceptors. Activation of these cells triggers a signaling cascade that propagates the transduced light information into several layers of processing, both at the retinal level and beyond (Pichaud et al., 1999). The detection and perception of color has been studied at many of these levels and in a variety of organisms, but we still do not understand many of the basic principles governing color vision (Conway et al., 2010).

**FIGURE 1:**
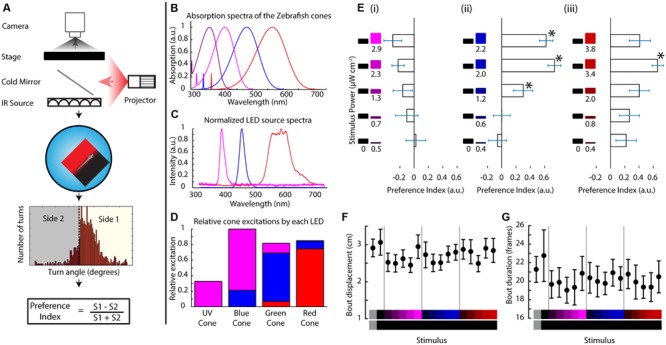
**Zebrafish larvae perform phototaxis and UV avoidance in a closed loop paradigm. (A)** Closed loop apparatus used in this manuscript. The fish is monitored in real time at ∼100 fps using a camera. The larva swims freely in a petri dish arena illuminated with infrared light. As the animal swims, its movements are tracked in real time and stimuli are projected from the bottom, aligned to the animal. After each experiment, the larval movement is segmented into individual turn events that are condensed into a preference index reflecting the stimulus choice of the animal. **(B)** Modeled absorption spectra of the larval zebrafish cone pigments, including the beta band of absorption. **(C)** Emission spectra of the LEDs used to project stimuli in this manuscript. **(D)** Theoretical relative excitation each LED elicits on each cone. The colors correspond to the LED colors in **(C)**. As mentioned, all cones are excited to an extent. **(E)** Larval turning direction preference indices for UV, blue and red stimuli against darkness. The stimuli are listed at the left side of each plot and preference index is expressed as the length of the horizontal bars (bars are mean ± SEM, *N* = 19 larvae from different clutches. Stars represent *p*-value < 0.05 in a paired bootstrap test comparing the lowest power level to the others). **(F,G)** average total displacement per bout **(F)** and duration of each bout **(G)** for each of the stimuli used above. Stimuli are indicated at the bottom with the color on each side represented vertically (points are mean ± SEM, fish are same as above).

Larval zebrafish have recently emerged as a model organism for the study of vision given their wide array of visually guided behaviors, such as the OMR (swimming in the direction of full field optic flow), OKR (eye movements for tracking moving objects), phototaxis (swimming from dark to light areas) and prey capture among others (Portugues et al., 2013). Their relevance in color vision research comes from their tetrachromatic retina, since aside from rods they have four types of cones that are morphologically distinct (Raymond et al., 1993). These cones are selective for four different wavelength ranges with varying degrees of overlap and functionally develop within the first 3 days post-fertilization (Branchek, 1984; Branchek and Bremiller, 1984; Schmitt and Dowling, 1999). Three of these cones have their center of absorption in the visible light range, namely at 570 (L cone), 480 (M cone), and 415 nm (S cone) and, interestingly, their fourth cone is UV sensitive, with an absorption spectrum centered at 362 nm (**Figure 1B**; Robinson et al., 1993; Govardovskii et al., 2000).

Given this potential for a rich color vision there have been a number of studies tackling the subject in adult fish. Three independent studies used training paradigms to evaluate color preferences of the fish, but found contradictory results and there was no control for stimulus luminance (Spence and Smith, 2008; Avdesh et al., 2012; Oliveira et al., 2015). Risner et al. (2006) and Thornberry et al. (2008) performed appetitive learning-based studies to test the wavelength discrimination capabilities of adult zebrafish, and to measure the behavioral spectral sensitivity function of the animal. By contrast, the effects of color stimuli on the behavior of larval zebrafish are relatively undescribed, despite a plethora of anatomical and electrophysiological studies that examine color processing circuitry in the larval retina, looking at connectivity (Branchek and Bremiller, 1984; Williams et al., 2010), functional properties (Robinson et al., 1993; Saszik et al., 1999; Cameron, 2002) and ontogeny (Branchek, 1984; Allison et al., 2010). The only behavioral study targeting color perception in the larva is centered on the OMR behavior, and it found that zebrafish larvae are red-green color blind to motion (Orger and Baier, 2005). Hence in our manuscript we contribute additional knowledge on larval zebrafish innate color sensing by measuring the behavioral responses of larvae to stimuli spanning the absorption range of their cones, and devoid of motion components. In particular, we will be relying on phototaxis, the tendency of the fish to move from areas of less light intensity to more light intensity. This behavior is not as robust as the OMR response, it has a dependency on the adaptation light level prior to the behavioral trial (Burgess et al., 2010) and it has a short memory-like component (Chen and Engert, 2014), but it does not involve a motion component in the visual field of the fish. Therefore it is ideal to test color stimuli in light of the findings by Orger and Baier (2005) outlined above. The neural circuitry required for this behavior has not been fully elucidated, but Burgess et al. (2010) proposed a mechanism in which the ON retinal pathway drives forward swims toward light sources while the OFF pathway drives turns against darkness. Given the latter work was performed using a relatively slow paradigm, Huang et al. (2013) developed a new phototaxis paradigm where a split field is projected below the fish, with one stimulus at each side. This field tracks the animal in real time using a feedback algorithm to update the projection position based on the location of the animal. Hence, it allows for very high temporal resolution in evaluating stimulus selection since each turn is a choice between the stimuli on one side vs. the other. This will be the main methodology for this study.

Although this manuscript examines the majority of the spectral range of zebrafish vision, the UV region of this spectrum is of particular interest given the diversity of ways UV vision has been utilized across the animal kingdom, and its unexplored usages by this model organism. Over the last 150 years, UV vision has been discovered and studied in many species, ranging from invertebrates such as ants (Lubbock, 1883) and bees (Chittka et al., 1994) to fish (Wolff, 1925), birds (Bennett et al., 1996) and mammals (Jacobs and Deegan, 1994). These studies found that UV signals are involved in many different behaviors, including mate choice, navigation and foraging. In fishes, there have been descriptions of mate selection via UV signals (Smith et al., 2002), UV-based foraging (Browman et al., 1994) and UV avoidance (Ylönen et al., 2004; Fukunishi et al., 2006). In zebrafish, Nava et al. (2011) probed the responses of adult zebrafish to UV light in a split tank assay and found an increase in escape responses, but the responses of larvae, which develop UV cones early and have a large peak in the UV region of their spectral sensitivity (Saszik et al., 1999), remain largely undescribed. Given the transparent nature of this animal and the presence of active UV cones in its retina, we hypothesized that larvae should react aversively to UV light sources.

Taking all of the above into account, we set out to describe the larval zebrafish’s innate responses to visible (defined as having a wavelength > 400 nm for the rest of the manuscript) and UV light using an existing phototaxis paradigm in closed loop.

## Materials and Methods

### Fish Rearing

Five–seven dpf male and female zebrafish larvae (*Danio rerio*, Hamilton, 1822) were bred in a 14:10 light/dark cycle at 28°C in 10 cm dishes. The strains used were TLAB (wild-type strain), *chokh*^-^*^/^*^-^ (strain that doesn’t develop eyes if homozygous for the mutation at this gene) and *Tg(sws1:CFP-TeNT)* (strain expressing tetanus toxin fused to Cyan Fluorescent Protein (CFP) in the UV cones only). All animal protocols were in accordance with NIH guidelines and the Harvard University IACUC.

### Closed Loop Phototaxis Assay

Zebrafish swam in a 10 cm petri dish while being tracked at 100 fps by a camera positioned above (AVT Pike F-032B, Allied Vision Technologies, Exton, PA, USA). The camera had an infrared filter mounted to avoid interference from the visible light stimuli. Visual stimuli were presented from below by a modified DLP projector (Lightcrafter Evaluation Module, Texas Instruments, Dallas, TX, USA) with LEDs centered at 606, 463, and 397 nm to match the red, green, and blue cones of the fish (Mouser Electronics, Mansfield, TX, USA). The projection is coupled to the IR illumination path by a long pass mirror (Edmund Optics, Barrington, NJ, USA). The tracking itself was performed via custom software developed in LabVIEW (National Instruments, Woburn, MA, USA). Briefly, each image is background subtracted, thresholded and then particles of a determined size are isolated and their coordinates and heading angle are recorded. Using this information the LabVIEW software also synthesized the stimuli to be shown to the fish. These stimuli consisted of a split field centered on the animal and aligned to its heading direction at every frame, prompting a choice between the sides at every turn event.

### Calculation and Measurement of Spectra

Spectra from the zebrafish cones were calculated based on the method and data described in Cameron (2002). Briefly, the calculation is based on the model developed in Govardovskii et al. (2000) for A1 based pigments (including both the alpha and beta bands) and the data compiled by Cameron (2002). The LED spectra were acquired using a CCS200 UV-Vis spectrometer, and their power was measured using a PM100D power meter with an S130VC sensor (all three from Thorlabs Inc., Newton, NJ, USA). Relative cone excitations were calculated by measuring the current sensed by the power meter sensor, weighting the normalized LED spectrum by it and then using the conversion of Watts/Amperes for each wavelength provided by the sensor manufacturer. Finally this power input was multiplied by the cone spectra and integrated to generate a relative excitation value.

### Trajectory Processing

The fish trajectories and heading angles were smoothed and then the position trace was segmented based on stretches of continuous velocity. These events were used to find the heading angle of the fish during the inter-event period, which allowed for calculation of the turning angle between events. These angles were used for calculation of preference indices per trial by thresholding the ones that represent turns (as opposed to bouts) and applying the following formula (Equation 1):

(1) $$\begin{array}{c} \mathrm{Preference}\;\mathrm{Index}\;=\;\frac{\left(S1\;-\;S2\right)}{\left(S1\;+\;S2\right)} \end{array}$$

These indices were then averaged across stimuli per fish, and final values were obtained by averaging across fish. Computations for this and subsequent analysis were performed in MATLAB (Mathworks, Natick, MA, USA). The custom software written can be obtained by contacting the corresponding author.

### Statistics and Error Analysis

Sample size (N) for every experiment in this study is defined as single larvae. Animals were pre-screened for phototaxis to a gray stimulus and only excluded if they failed to show this behavior (pre-established criterion). Standard errors were determined by performing a bootstrap with the preference indices from each fish and 1000 iterations using the mean of these indices. The same process was performed comparing the differences between the preference indices of the stimuli of interest to test for significance at a critical value of 0.05 (two-tailed). We chose to use bootstrapping since there is no indication the data will be normally distributed, and hence opted for this non-parametric technique.

### Variable Contrast Experiments

The paradigm used in the rest of the manuscript was modified to change contrast as a function of the fish turning direction. In particular, the animal is presented with a split field stimulus in a closed loop fashion. If the animal turns toward the light side of the stimulus the contrast between the two sides is decreased. If in turn the animal swims toward the dark side then the contrast of the stimulus is increased. For determining the contrast change there was an angle change threshold of 10 degrees within a sliding window of 1 s. At each threshold crossing the intensity of one side was increased by about ∼5 nW cm^-2^ while the intensity of the other side was decreased by the same amount.

## Results

### Construction of a Visual Stimulation Instrument for Free Swimming Larval Zebrafish

In this assay a stimulus is projected below the fish on a closed loop so that it stays centered on, and aligned to the animal. The stimulus shows a left/right divided field, prompting the fish to choose between the two sides whenever it turns (**Figure 1A**). For this manuscript, we modified a projector to show stimuli centered on the fish’s L, M, and S cone’s absorption spectra while still partially overlapping with the UV cone (LEDs with emission spectra centered at 606, 463, and 397 nm, **Figure 1C**). Although our three-channel projector is not capable of isolating stimulation to a particular cone type, it is still highly selective and manages to activate all four cones to different degrees as shown in **Figure 1D** (centering activation on the L, M, and S + UV cones, respectively). Hence from here on out when referring to the red, blue or UV stimulus we will be referring to the relative excitations shown in **Figure 1D** or their linear sum if using a combined color stimulus (detailed cone excitations can be seen in Supplementary Figure S1A).

### Zebrafish Larvae Innately Swim Toward Visible Light and Away from Ultraviolet Light

When the fish (TLAB strain) was presented with varying levels of each LED on one side and darkness on the other, it swam preferentially in the lit direction for the red and blue stimuli (what we will define as phototaxis from here on), but it swam weakly toward darkness when shown the UV stimuli as can be seen in **Figure 1E** (values shown are averaged preference indices ± SEM). Both of these responses were observed in an intensity dependent manner across 19 fish. We also looked at the stimulus effects on swimming parameters. In particular, we looked at the features of every individual movement event of the animal (termed “bout”). As shown in **Figures 1F,G**, there was no change in the total displacement per bout or in the duration of the bouts, meaning the preference change we observe is due mainly to turn direction without influences from other swimming parameters (see also Supplementary Figure S1B). This is the first demonstration of an innate motor response to UV light in the larval zebrafish.

### Phototaxis and UV Avoidance Interact to Determine Turning Direction

**FIGURE 2:**
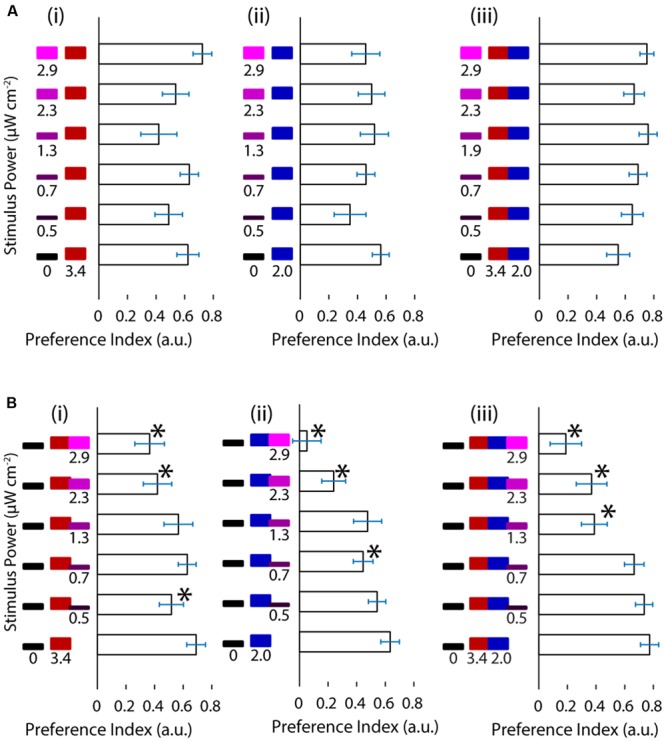
**Ultraviolet light is avoided in a manner indistinguishable from darkness and it competes with phototaxis. (A)** Preference shown by the larvae for varying levels of UV stimuli vs. red, blue or both red and blue stimuli. **(B)** Preference of the larvae for darkness vs. red, blue or red and blue stimuli with increasing levels of the UV stimulus. (bars are mean ± SEM, *N* = 21 for the A panels, *N* = 20 for the B panels, both with fish from several clutches. Stars represent a *p*-value < 0.05 in a paired bootstrap test comparing the lowest power value to the others).

We wanted to ask whether the relationship between UV avoidance and phototaxis can be revealed by the combination of the stimuli involved in each of the behaviors. First we asked whether UV avoidance could act in cooperation with phototaxis to drive the TLAB animals away from UV and toward visible stimuli, hence amplifying the turning preference. We presented the animal with increasing levels of the UV stimulus on one side (maximum at 2.9 μW cm^-2^) and a constant, highly attractive level of the visible stimulus on the other (maximum at 5.4 μW cm^-2^, **Figure 2A**). At the light intensities used the response did not behave as cooperation between avoidance against one side and phototaxis toward the other. In particular, the fish failed to show any modulation in their response with increasing levels of UV light.

Given these results we asked the opposite question, namely, how does the fish respond when UV avoidance and phototaxis are pitted against each other? To achieve this, we designed a stimulus featuring darkness on one side of the fish and a mixture of the UV and visible stimuli on the other. We held the visible stimulus constant and increased the intensity of the UV stimulus as above. As shown in **Figure 2B** the preference of the animal for the light side decreased as a function of the intensity of the UV stimulus. This was observed across the different visible light stimuli and with different slopes, showing that UV avoidance can compete against phototaxis. Despite the clear effects in turning preference, there was no change in the fish’s swimming parameters as a function of stimulus (Supplementary Figure S2).

Up to this point we have shown that under the conditions in this study the responses to UV and visible stimuli can interfere destructively but not constructively.

### UV Avoidance Is Mediated by Functional UV Cones in the Retina

**FIGURE 3:**
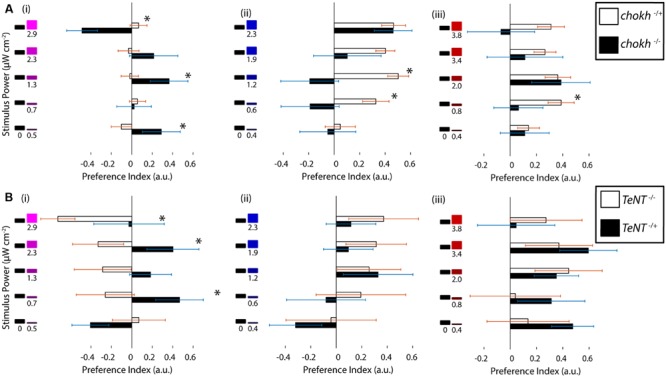
**Ultraviolet avoidance is mediated by a visual process, and it depends on the presence of functional UV cones. (A)** Preferences of eyeless *chokh* mutant fish and their wildtype siblings for the UV, blue and red stimuli vs. darkness. White bars show the wild type animal results and black bars the blind mutant results (*N* = 18). **(B)** Preferences of *sws1*::TeNT-CFP transgenic fish with inactive UV cones vs. their wildtype siblings. The stimuli consisted of darkness vs. varying intensities of the UV, blue or red stimuli as in part A and **Figure 1**. The white bars show the wild type fish results and the black bars show the transgenic animal results (*N* = 8, bars in both experiments are mean ± SEM with animals from different clutches. Stars represent a *p*-value < 0.05 in an unpaired *t*-test comparing the two conditions).

Zebrafish have an extremely rich array of non-visual opsins, expressed in a number of brain regions and also outside the brain (Kojima et al., 2000; Kokel et al., 2013). Some of these opsins – such as melanopsin (Matos-Cruz et al., 2011) and UV opsin itself (Davies et al., 2015) – have been shown to be behaviorally relevant. Hence, we asked whether the observed UV avoidance was mainly visually driven or not, especially given the results from Fernandes et al. (2012) on a phototaxis behavior mediated by these opsins. To this end we performed phototaxis experiments in *chokh*^-/-^ mutants, which lack eyes due to a mutated homeobox protein (Loosli et al., 2003), and we compared these to their wild type siblings. As shown in **Figure 3A** the mutant fish show an extremely erratic response while their wild type siblings exhibit normal phototaxis. To our surprise some preference indices in the mutant fish were fairly high, but this is probably due to the lower bout frequency in this group, and is confirmed by the lack of a trend for any of the stimuli in the series. Aside from the bout frequency, all the other bout parameters are very similar between the mutant and wild type animals (Supplementary Figure S3A).

Given our behavioral results and the above experiments with the *chokh* mutant, we hypothesized that the UV cone in the eye must have a role in the response. To test this idea we used a transgenic animal with inactive UV cones. In this transgenic, Yoshimatsu et al. (2014) inserted a cassette containing the *sws1* promoter [normally driving expression of the UV opsin (Takechi et al., 2003; Luo et al., 2004)], followed by a fusion of Tetanus toxin and the CFP. Expression of this fusion renders the UV cones inactive and also allows for screening of the individuals with successful transgenesis (Yoshimatsu et al., 2014). When the transgenic fish were tested in our paradigm, and compared with their wild type siblings, we observed that the responses to red and blue stimuli were similar between groups but the responses to UV stimuli were abolished in the transgenic, as depicted in **Figure 3B** (other swimming parameters remain constant, as shown in Supplementary Figure S3B). Finally, the response to UV stimuli seems to have an inverse sign, although this was found to be not significant.

In summary this evidence points to UV avoidance as a visually guided behavior that depends on the presence of functional UV cones.

### Avoidance of the UV Stimuli and Preference for the Visible Stimuli are also Affected by Stimulus Contrast

**FIGURE 4:**
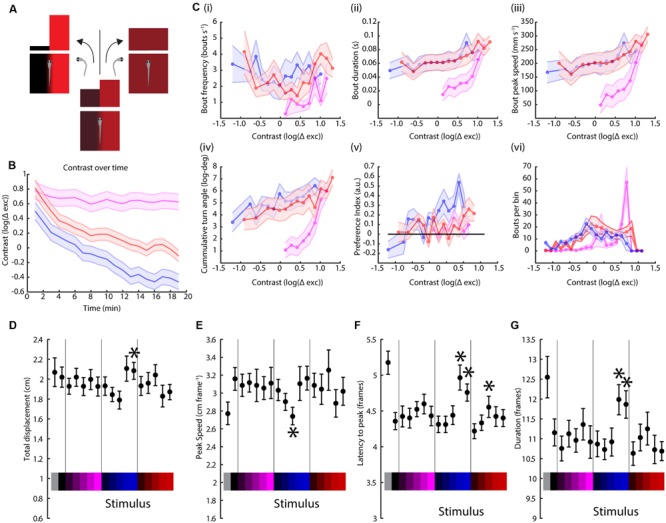
**Contrast variation but not full field stimulation have wavelength-dependent effects on swimming parameters. (A)** Schematic of the modified paradigm used. Briefly, the animal is presented with a split field stimulus as in the figures above, but now the contrast between the two sides decreases if the fish turns toward the lighter side (higher irradiance) and increases if the turn is toward the darker side (lower irradiance). **(B)** Contrast of each stimulus (UV, blue and red, colored as before) over time, averaged over two trials per experiment and 14 TLAB fish (the lines are mean ± SEM). The units are the logarithm of the total cone excitation difference between the two sides. **(C)** Modulation of (i) bout frequency, (ii) duration, (iii) peak speed, (iv) turn angle, (v) preference index, and (vi) number of bouts by stimulus contrast (points are averages across 14 animals ± SEM). **(D–G)** Dependency of bout displacement **(D)**, speed **(E)**, latency **(F)**, and duration **(G)** on full field stimulation (points are mean ± SEM, *N* = 23, stars represent a *p*-value < 0.05 on a paired bootstrap test against the first power level).

Visual systems tend to focus on differences rather than absolute levels (Heeger et al., 1999; Lee et al., 2002), so we attempted to measure the effect of stimulus contrast, rather than absolute intensity, on phototaxis. For this, we devised a modified paradigm to cover a range of different contrasts as a function of the turning behavior of the animal. In this experiment, the TLAB larvae are also presented with a split field stimulus, but if the fish turns toward the lighter (higher irradiance) side of the stimulus, the contrast between the two sides is reduced [by increasing the intensity of the darker side (lower irradiance) and reducing the intensity of the lighter side]. If the fish swims toward the darker side then the contrast is increased (**Figure 4A**). We probed the fish responses to the red, blue, and UV stimuli in this paradigm and the results can be observed in **Figure 4B**. As shown, the animal successfully decreased the contrast during the red and blue stimuli by swimming toward the lighter side, but instead managed to keep the contrast high during the UV stimulus, which can only be achieved by swimming mainly toward the darker side while avoiding the lighter side. As shown in **Figure 4C**, we also observed distinct differences in a number of swimming parameters as a function of contrast. In particular, for bout frequency, bout duration, bout peak speed and cumulative turning angle the fish seemed to show a linear, very gradual response to contrast under the red and blue stimuli but this turned into a much steeper response under the UV stimulus.

### Full Field Stimulation Fails to Elicit Wavelength-Specific Responses

An unanswered question from the experiments above is whether there are any discernible effects from a full field stimulus instead of a split field, since this kind of modulation will play a role in the interpretation of our preference index data. As expected, there was no turning angle preference observed in this assay (as shown in Supplementary Figure S4). **Figures 4D–G** indicate there is weak light intensity-dependent modulation in bout duration, peak speed, latency to peak speed and distance traveled. More importantly, there was no observed wavelength modulation of the stimulus, which is consistent with the observations from the same swimming parameters for the split field stimuli, as shown in **Figure 1** and in the Supplementary Information.

## Discussion

We have shown that zebrafish larvae swim toward visible light stimuli and away from UV stimuli in a closed loop split-field paradigm. This preference is expressed mainly via turning direction with no discernible changes in other swimming parameters as a function of the stimulus. The UV avoidance behavior interacts negatively but not cooperatively with phototaxis, and relies on the visual system, and the UV cone in particular, to function. Finally, phototaxis to visible stimuli seems to be a shallow function of contrast while UV avoidance shows a sharp change.

### Functional Relevance of UV Light Detection

Ultraviolet vision is present across the animal kingdom (Losey et al., 1999; Yokoyama and Shi, 2000; Shi et al., 2001), serving a wide range of roles such as food and mate selection. Fish have been shown to use UV light inputs in a variety of ways, including detection of UV pigmentation for recognition of conspecifics, enhanced foraging and deployment of protective pigments (Ward et al., 2008; Sabbah et al., 2010). In zebrafish it has been posed that UV detection might be used to avoid photodamage by mediating the deployment of pigment granules called melanosomes (Mueller and Neuhauss, 2014). This makes ontological sense since larval zebrafish have been shown to be particularly prone to damage from UV radiation due to their transparency (Sandrini et al., 2009) and are generally sensitive to low wavelength illumination (Villamizar et al., 2014). The behavior we describe in this manuscript is consistent with this hypothesis as it allows the animal to remove itself from regions of high UV radiation. Notably, this evidence still does not explain the positioning of the UV cones in a potentially image forming role, pointing to additional, undescribed functions that will require further investigation.

### Interaction between UV Avoidance and Phototaxis

In our study, phototaxis and UV avoidance occur, and interact, competitively rather than cooperatively. One hypothesis explaining this one-sided interference stems from the ecology of the zebrafish: larvae live at low depth with varying levels of turbidity (Spence et al., 2008), which potentially exposes them to high levels of UV irradiation (Booth and Morrow, 1997). At the same time, the larvae rely heavily on visual input to find food and avoid predators. Therefore, a balance needs to exist between avoiding UV irradiation yet remaining in areas with enough light for their visual system. This would explain why, when avoiding UV or darkness, the response is to approach a UV-free source of light, but when the light source has UV radiation then the preference decreases as a function of the intensity of the UV component. This also answers why UV avoidance is so weak when presented on its own against darkness. However, this weak avoidance could alternatively be explained by some residual activation of circuits for phototaxis during presentation of our UV stimuli, which, in addition to stimulating UV cones, drive the S (415 nm) and M (480 nm) cones (see **Figure 1D**). This would not be observed with a lower wavelength (∼365 nm) source like the one used in Nava et al. (2011) to elicit escapes in adults, since the main cone being stimulated would only be the UV cone. Along these lines, the weak avoidance of UV toward darkness may also stem from the power output of our light source, which may not maximally activate the UV cones. In this case, a more powerful source might be able to generate a stronger avoidance response during presentation of UV light. Yet another alternative is that the larvae use UV detection for polarization vision, explaining the weak responses by the lack of polarization in our stimuli. Polarization has been described as a visual modality in other aquatic animals (Hawryshyn and McFarland, 1986) but there are no reports of the presence or absence of this capability in zebrafish. Finally, it should be noted that in this setup we only consider possible lateral responses to UV light, and neglect any potential swims toward or away from the surface of the water. Given the increased scattering of UV light relative to other wavelengths in deeper water (Booth and Morrow, 1997), the presence of UV light may spur larvae to move toward lower depths. This is a possibility that cannot be assessed with our paradigm but that could drive future experiments in 3D arenas.

### UV and Color Vision Circuitry

Our results with the tetanus toxin-expressing animals point to a retinofugal pathway including UV cone signals contributing to the UV avoidance behavior. Studies by Saszik et al. (1999) and Bilotta et al. (2005) measured the responses elicited by UV illumination in the ERG of the larval zebrafish. They report that the “a” wave component of the ERG in response to UV stimuli is distinct from the response obtained from stimulating with higher wavelengths. This difference does not originate at the photoreceptor level, suggesting the transduction of UV information into the rest of the zebrafish brain. From the perspective of connectivity, Li et al. (2012) report that there are likely no cone type-exclusive bipolar cells in the adult retina, and hence it is also unlikely there are cone-exclusive retinal ganglion cells sending these signals out of the retina. This is consistent with a model where UV radiation is a very salient stimulus for the animal, but is not detected in isolation. Instead, and under the context of this behavior, it is probably represented in the most relevant way given lighting in the wild, namely as a negative component of the overall light input as explained above. Very little is currently known about color signal processing in the larval zebrafish beyond the retina, making the actual neural location and basis for the computation of turn direction from the chromatic inputs in the brain open for speculation. Candidate regions include the retina itself, the arborization fields of the optic tract, the optic tectum and downstream, undescribed areas.

The next step in deciphering this circuit will require the use of techniques to identify the regions and cell types involved, past the information that can be gained through behavior. Given the advances made during the last decade in the field of deep brain 2-photon calcium imaging in zebrafish larva (Ahrens et al., 2013a,b; Portugues et al., 2013), this seems like the ideal avenue to take for furthering knowledge in this system and should spearhead future studies in the subject of UV and color vision.

## Author Contributions

DG-N conceived the study, carried out the experiments and helped design them, wrote the manuscript and conceived the figures. FE helped design the experiments and helped in drafting the manuscript.

## Conflict of Interest Statement

The authors declare that the research was conducted in the absence of any commercial or financial relationships that could be construed as a potential conflict of interest.

## Abbreviations

dpf: days post fertilization
ERG: electroretinogram
fps: frames per second
OKR: opto-kinetic response
OMR: opto-motor response
SEM: standard error of the mean
UV: ultraviolet
