# Supplementary material for: Properties of the Visible Light Phototaxis and UV Avoidance Behaviors in the Larval Zebrafish
Source: Front Behav Neurosci. 2016 Aug 19;10:160. doi: 10.3389/fnbeh.2016.00160 (PMC4990545; doi:10.3389/fnbeh.2016.00160)
Supplement: Supplementary file 1 [file Data_Sheet_1.PDF]

Properties of the visible light phototaxis and UV avoidance behaviors in the larval zebrafish

Drago A. Guggiana-Nilo1,2 and Florian Engert2*

Supplementary figures:

Supplementary figure 1: Cone excitations for each stimulus and bout parameters for Figure 1

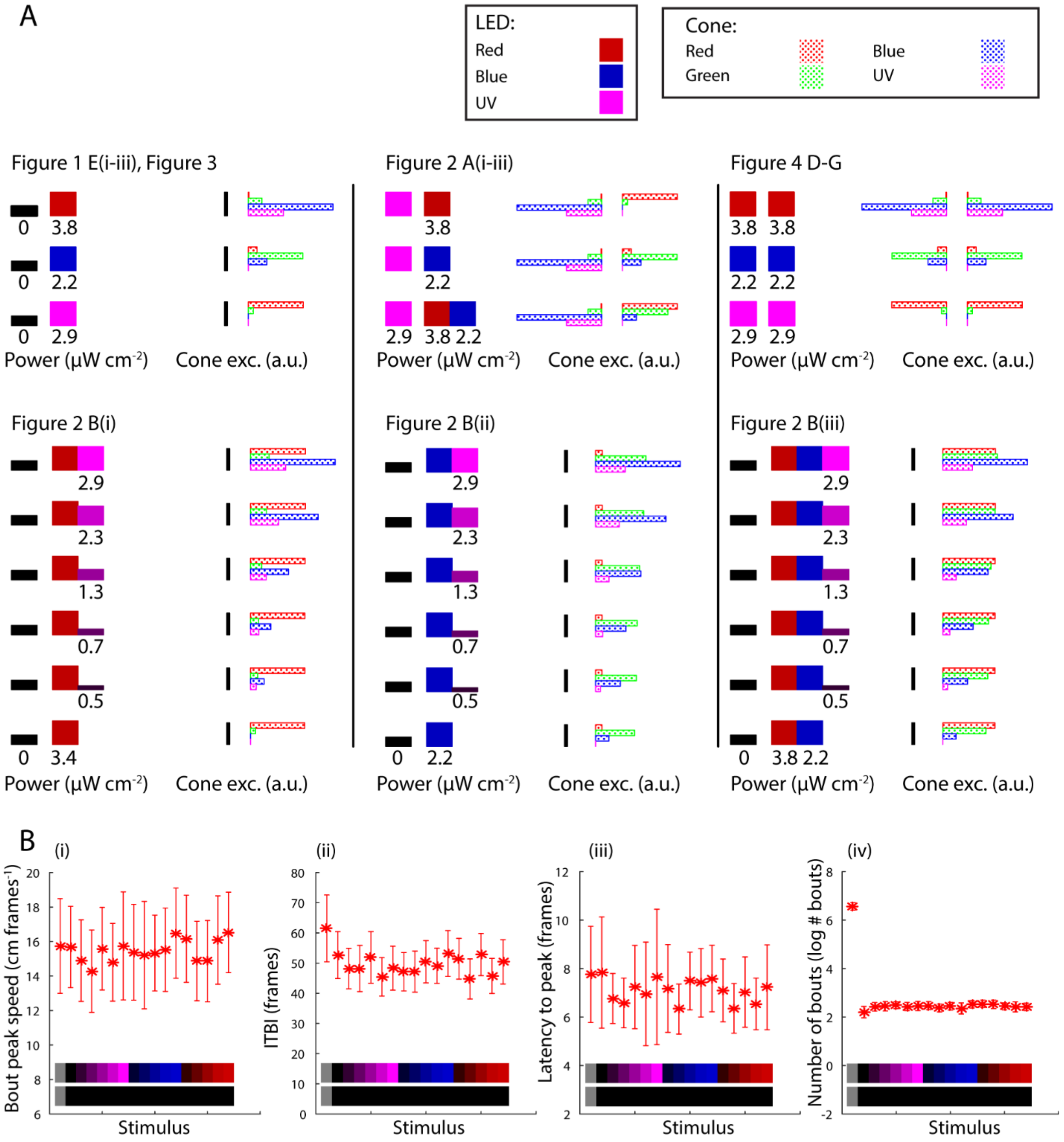

A: Cone excitations from each of the stimuli used in the study. The solid columns on the left show the stimuli as they are shown in the respective figures (indicated at the top of each panel), including the power density measured at the sample (red, blue and magenta bars represent the 3 LEDs used in the study). The spotted columns on the right show the relative excitation to each one of the zebrafish cone types from these stimuli. The L (red), M (green), S (blue) and UV cones are represented by the red, green, blue and magenta bars. Black bars represent no stimulus. These excitations were calculated by convolving the emission spectra of each LED at each intensity by the modeled absorption curves of each cone as shown in Figure 1 B. **B(i-iv):** Bout parameters for the phototaxis experiments shown in Figure 1 Ei-iii. There was no apparent trend in any of them, either depending on stimulus or light intensity (gray represents the rest stimulus and has more bouts because there is a rest in between every trial). Values are mean±s.e.m..

Supplementary figure 2: Average bout parameters for the larvae during each stimulus in Figure 2.

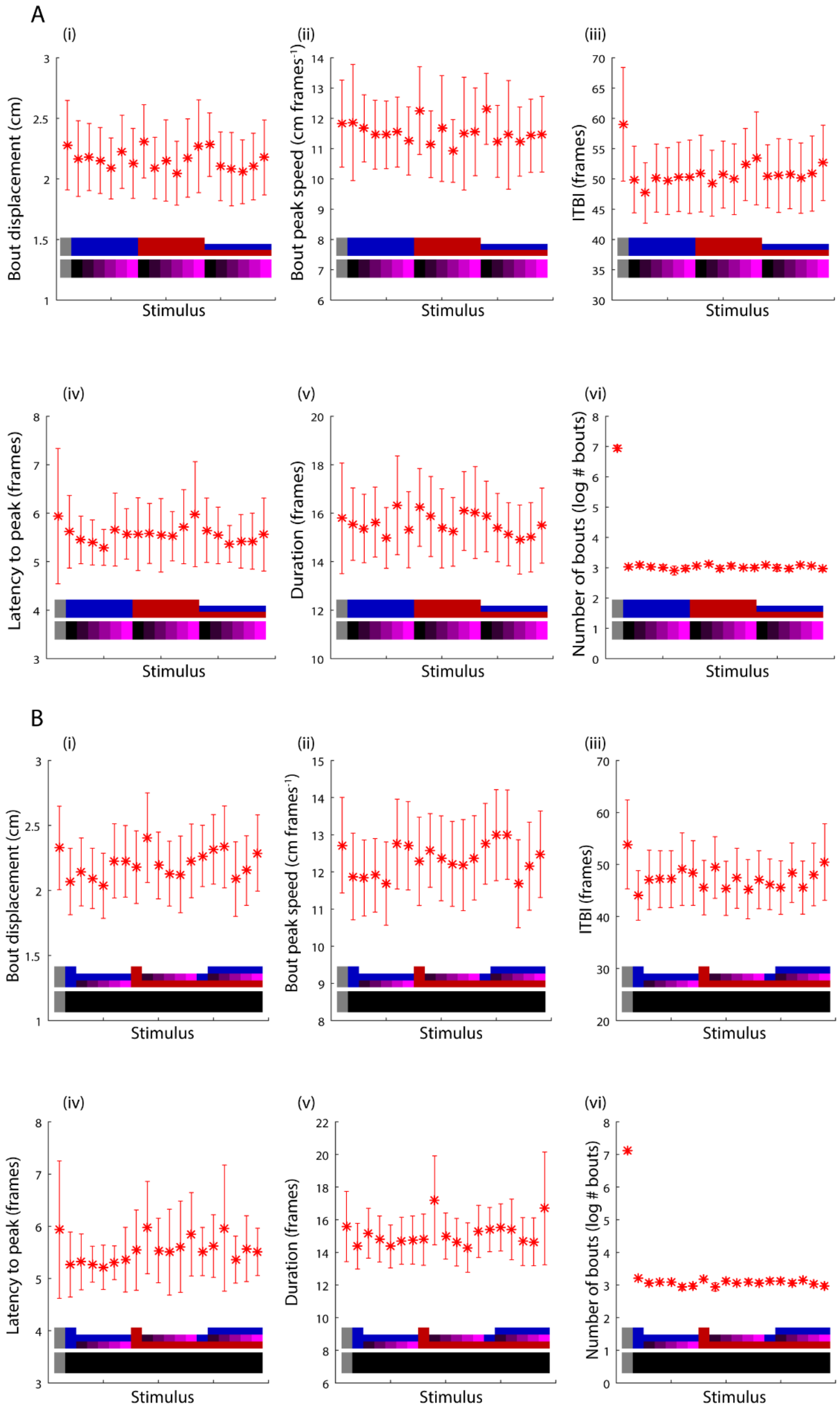

A(i-vi): Displacement per bout, Bout peak speed, InTer-Bout Interval, Latency to peak speed, Bout duration and Number of bouts averaged for each stimulus in Figure 2A. Gray represents all LEDs engaged on both sides (which is the rest stimulus, shown in between every other stimulus and hence showing more bouts than the others), black is LEDs off on both sides, and the other stimuli all represent a color on one side and black on the other side. As shown, there is no discernible modulation of either of this parameters based on stimulus. **B(i-vi):** Same kinematic parameters outlined for panel A but for the experiments in Figure 2B(i-iii). The stimuli showing the replacement of darkness with UV are shown in A and the stimuli that merge in UV in the color side are shown in B. There is not significant modulation of the response, either from stimulus or light intensity.

Supplementary figure 3: Average kinematics of the *chokh* and *TeNT* larvae during experiments.

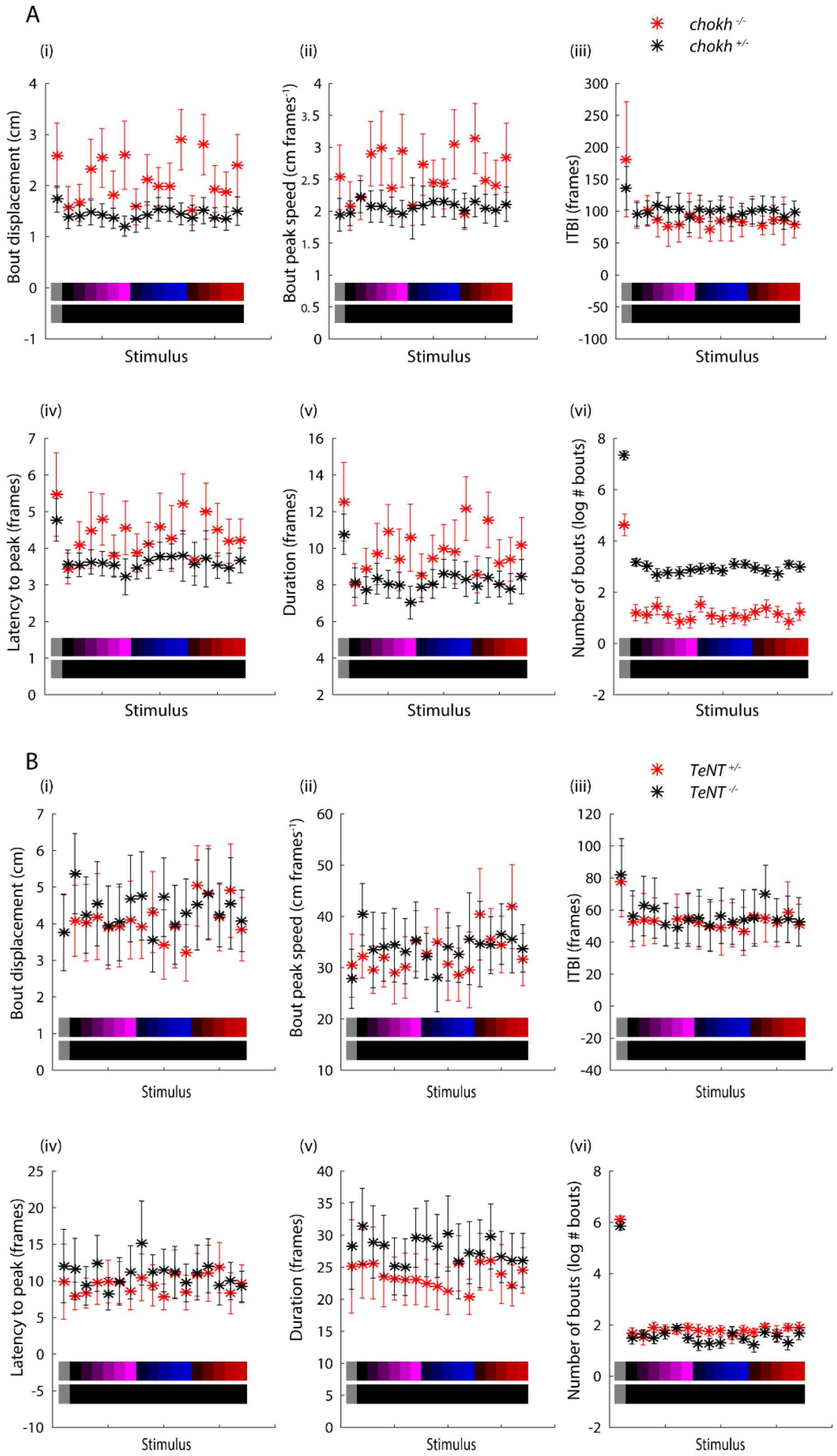

A(i-vi): Displacement per bout, Bout peak speed, Interbout interval (ITBI), Latency to peak speed, Bout duration and Number of bouts averaged for each stimulus in Figure 3A. The red symbols show the mutant fish and the black symbols the wild type siblings. Although the wild types show more bouts in average, the bout statistics for the fish seem very similar. **B(i-vi):** same parameters as in A for the TeNT larvae. Red symbols show the TeNT-expressing larvae and black symbols their wild type siblings. No discernible difference was observed between conditions or between stimuli.

Supplementary figure 4: Preference index shown by larvae during full field stimuli

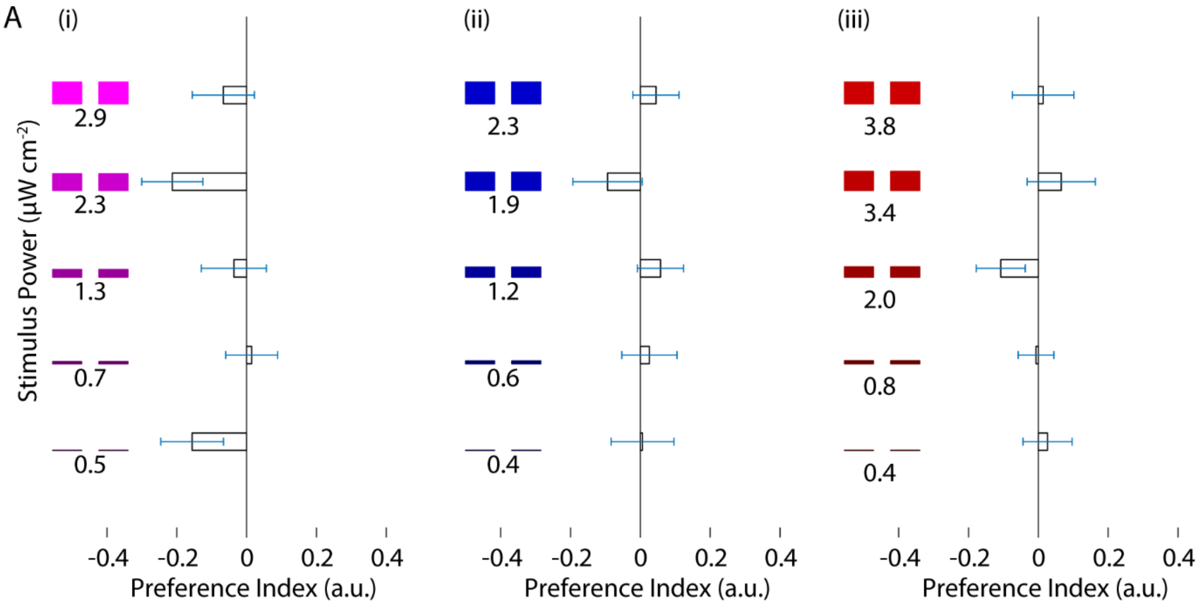

A(i-iii): preference indices calculated from the full field stimuli experiments. This shows the expected response of no preference given there is no difference between the sides.
